# Risk Assessment and Recommended Approaches to Optimize Infection Control and Antibiotic Stewardship to Reduce External Ventricular Drain Infection: A Single-Center Study

**DOI:** 10.3390/antibiotics13111093

**Published:** 2024-11-17

**Authors:** Jozsef Kelemen, Marton Sztermen, Eva Dakos, Gergely Agocs, Jozsef Budai, Jozsef Katona, Zsuzsanna Szekeressy, Laszlo Sipos, Zoltan Papp, Mate Bata, Janos Karczub, Mate Korompai, Zsuzsanna A. Dunai, Bela Kocsis, Dora Szabo, Lorand Eross

**Affiliations:** 1 Deparment of Neurosurgery and Neurointervention, Semmelweis University, 1085 Budapest, Hungary; kelemen.jozsef.arpad@semmelweis.hu (J.K.); sztermen.marton@semmleweis.hu (M.S.); dakos.eva.krisztina@semmelweis.hu (E.D.); budai.jozsef.andras@semmelweis.hu (J.B.); katona.jozsef@semmelweis.hu (J.K.); sipos.laszlo.kornel@semmelweis.hu (L.S.); papp.zoltan.attila@semmelweis.hu (Z.P.); bata.mate@semmelweis.hu (M.B.); karczub.janos@semmelweis.hu (J.K.); korompai.mate@semmelweis.hu (M.K.); eross.lorand@semmelweis.hu (L.E.); 2 Department of Biophysics and Radiation Biology, Semmelweis University, 1094 Budapest, Hungary; agocs.gergely@semmelweis.hu; 3 Gyula Nyírő National Institute of Psychiatry and Addiction, 1135 Budapest, Hungary; szekeressyzs@nyiro-opai.hu; 4 HUN-REN-SU Human Microbiota Research Group, 1052 Budapest, Hungary; zsuzsanna.dunai@gmail.com; 5 Institute of Medical Microbiology, Semmelweis University, 1089 Budapest, Hungary; kocsis.bela@semmelweis.hu

**Keywords:** central nervous system (CNS), hospital-acquired infection, neurosurgery, external ventricular drain (EVD), EVD infection, post-neurosurgical meningitis

## Abstract

**Background:** An external ventricular drain (EVD) is used to release elevated intracranial pressure by draining cerebrospinal fluid (CSF) from the brain’s ventricles. The establishment of an EVD is one of the most commonly performed neurosurgical procedures to treat intracranial pressure in patients. Nevertheless, infections are very frequent complications. Identifying the risk factors for EVD-related infections is a key to improving patient safety and outcomes. **Methods:** We conducted a retrospective, single-center study of patients who underwent EVD implantation between January 2022 and March 2024. Patients were classified into infected and non-infected groups based on their clinical symptoms, as well as laboratory and microbiological results. Patient characteristics and possible risk factors for infection were compared between the two groups. **Results:** In total, 123 patients treated with 156 EVDs were included in this study, with a mean age of 55.8 (range: 25–84) years. EVD-associated infections were observed in 37 patients (30%). We found no significant association between infection risk and patient characteristics, including gender, primary diagnosis, craniotomy, or immunosuppression. There was no significant difference in terms of EVD insertion, i.e., whether the insertion took place in the operating room (OR) with antibiotic prophylaxis or outside the OR with no periprocedural antibiotic treatment. However, within the intensive care unit (ICU), EVD infection was much lower (13%) if EVD insertion took place in a single-bed room compared to multiple-bed room insertions (34%). Furthermore, there were significant differences in terms of the duration of first EVD (both single and multiple catheterizations) (*p* < 0.0001) and the total catheterization time (*p* = 0.0001). Additionally, there was a significant association with patient days in the ICU and EVD catheterization. **Conclusions:** Revisiting infection control measures is necessary, with special attention to the replacement of EVDs in single-bed ICU rooms, to introduce antibiotic prophylaxis in the ICU. Minimizing unnecessary EVD manipulation during catheterization is crucial in order to decrease the risk of EVD infection.

## 1. Introduction

An external ventricular drain (EVD) is used in neurosurgery to treat various conditions. In neurosurgical practice, the use of EVDs is essential to monitor and reduce intracranial pressure in conditions such as hydrocephalus, traumatic brain injury, subarachnoid hemorrhage (SAH), and intraventricular hemorrhage (IVH). Despite the fact that their use in these clinical conditions is life-saving, the use of EVDs is associated with the risk of developing a number of complications, with infection being potentially the most dangerous. EVD-related infections, including ventriculitis and meningitis, are associated with longer hospital stays, increased healthcare costs, and higher morbidity and mortality rates [1,2,3,4].

Infections can lead to devastating consequences, such as brain abscess or subdural empyema, underscoring the risk factor of infection and the need for careful consideration of prevention strategies. Although the complications of EVDs, such as bleeding, catheter malposition, and catheter occlusion, are well known, infections represent the greatest clinical challenge. Infection control for EVDs is critical due to the high rates of infection associated with their use. The rate of EVD-related infections varies widely, ranging from 0% to 45%, depending upon factors such as patient population, catheter management protocols, and even the definitions used to define infections and the criteria used to confirm the diagnosis of infection [1,5].

Several factors have been implicated in the development of EVD infections, including prolonged catheterization, frequent cerebrospinal fluid (CSF) sampling, catheter manipulation, and insufficient tunneling of the catheter [1,2,3,4,5,6,7,8,9,10,11,12,13,14,15,16,17].

To date, limited data are available on the specific role of EVD placement and prolonged catheterization in increasing the risk of infection, particularly in centers where antibiotic prophylaxis is not consistently used. The aim of our study was to examine risk factors for EVD-related infections using the PECO (Population–Exposure–Comparisons–Outcome) framework. We analyzed the patients of a single-center cohort (Population) who underwent EVD placement (Exposure) in different settings (Comparisons), comparing those who developed infection vs. those who did not (Outcome).

## 2. Results

**Figure 1:**
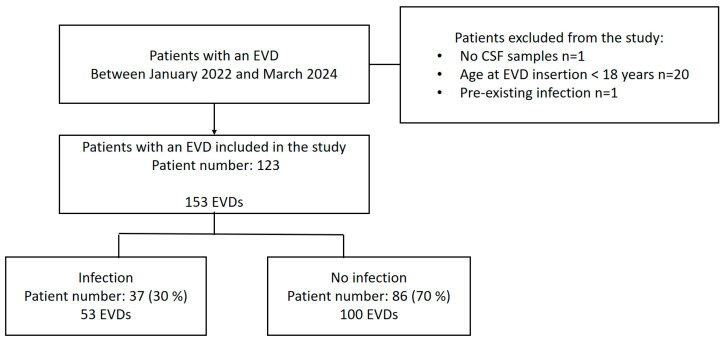
Flowchart of patient inclusion in this study, detailing EVD catheterization and infection status.

Overall, we analyzed data on 153 EVDs inserted in 123 patients for 2107 EVD days. A flowchart of the patient characteristics included in this study is shown in Figure 1. We observed similar distribution patterns of sex for patients receiving EVDs, with 43.24% of patients being male in the infected group and 46.51% in the non-infected group, with no statistical differences (*p* = value 0.77, Fisher’s exact test; Table 1). The age range was 25 to 84 years, and the median age was 54 years (Table 1). The ratio of women in the infected group was 56.76%, and it was 53.49% in the non-infected group. The patient characteristics and differences between patients with and without EVD infection are shown in Table 1.

### 2.1. Impact of Comorbidities on the Risk of EVD Infection

The indications for EVD placement were SAH in 98 patients (79.67%), ICH in 25 patients (20.32%), other intracranial bleeding in 3 patients (2.44%), brain occlusion or stenosis in 12 patients (9.76%), and, finally, tumors in 12 patients. Both in the EVD-infected and non-infected groups, the most common primary diagnosis was SAH (32 patients, 86.49% vs. 66 patients, 76.64%), the difference was not significant (*p* = 0.5229 using a resampling method; Table 1). Seventy-two patients (58.53%) were intubated at the time of EVD insertion, with no difference between the infected and non-infected groups (*p* = 0.5955, resampling). Ten patients were immunocompromised (8%), five patients (13.51%) in the infected group and five patients (5.81%) in the non-infected group (*p* = 0.4737, resampling). In addition, diabetes mellitus was present in six patients (16.22%) in the infected group and nine patients (10.47%) in the non-infected group, without a significant difference (*p* = 0.5246, resampling). The number of EVDs placed in patients with craniotomy was 8 (21.62%) in the infected group and 27 (31.34%) in the non-infected group. We found no significant difference between the tested patient characteristics in the infected and non-infected groups.

### 2.2. Impact of Operating Conditions on the Risk of EVD Infection

Only two patients received their first EVD in another hospital, and one of them developed an EVD infection. Ninety-nine patients (80.48%) received their first EVD at the ICU, and twenty-one patients (19.51%) received it in the operating room (OR) together with other interventions (Table 2). Patients with EVD insertion in the OR received perioperative antibiotic prophylaxis as part of the surgical protocol.

**Figure 2:**
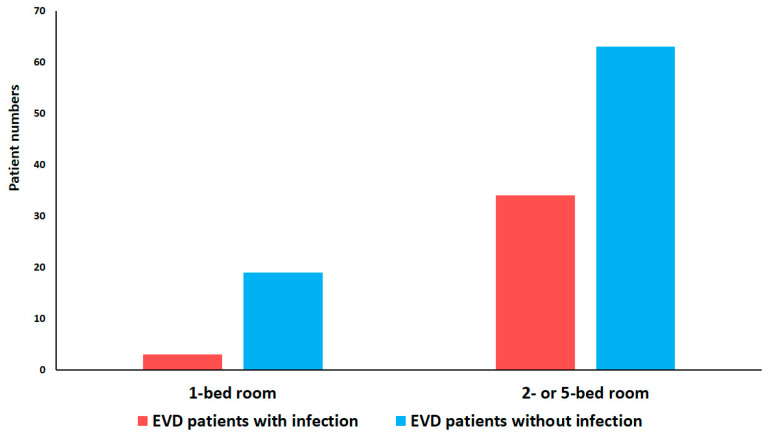
The bed counts in ICU in the infected and non-infected groups of EVD patients.

In the non-infected group, 67 cases (78%) were admitted to the ICU, and 18 cases (21%) had to go through surgery, while in the infected, group 31 cases (84%) were admitted to the ICU and 5 cases (14%) to the OR (Table 2). Although no difference can be observed in terms of whether the EVD was placed in the OR (*p* = 0.5384 resampling) or in the ICU (*p* = 0.5667; resampling), a difference can be observed in the various rooms within the ICU. If the placement occurred in a single-bed room, the infection rate was only 13%, compared to the 34% infection rate when the patients were housed in two-bed or five-bed rooms (Figure 2), reaching significant difference. Furthermore, a significant association (*p* < 0.0001, Welch test) could be observed between patient days in the ICU and EVD catheterization.

### 2.3. Impact of EVD Charateristics on the Risk of EVD Infection

An EVD infection is influenced by the catheter characteristics as well. The catheter characteristics and differences between patients with and without EVD infection are shown in Table 2.

There was a significant difference (*p* = 0.0211, Fisher’s exact test) between the infected and non-infected group with regard to the multiplicity (i.e., single or multiple) of catheterization. Furthermore, we observed a significant association between the total catheterization days and catheterization infection (*p* = 0.0001, Welch test).

**Figure 3:**
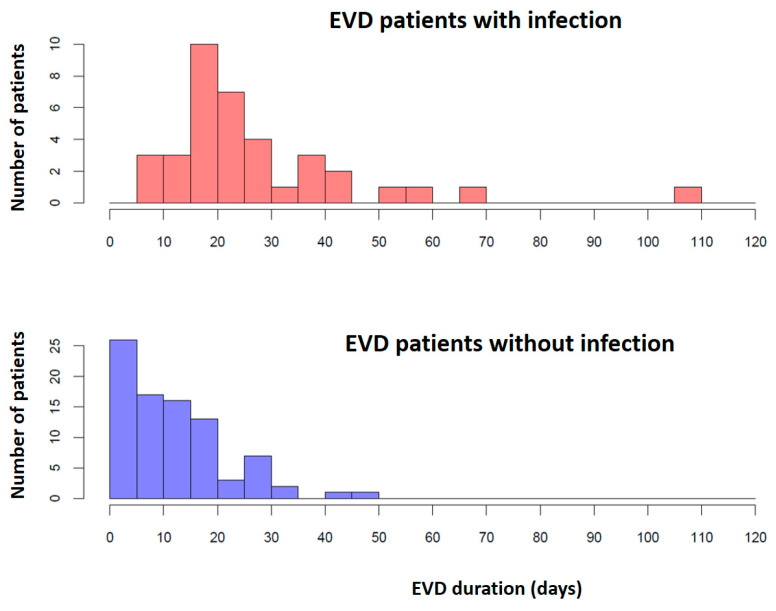
Infection in relation to duration of EVD.

In the non-infected group, the number of patients decreased as the number of EVD days increased, while the infected group exhibited a marked increase in the number of patients on day 15, after which a gradual decrease was observed (Figure 3). An ROC analysis yielded 14.5 EVD days as the optimal cut-off point for the dichotomization of the EVD duration with regard to infection outcome.

By comparing the number of total EVD reinsertions needed per patient, we observed a significantly increased number in the infection group, with 12 patients (32%), compared to the non-infection group, with 11 patients or 13% (*p* = 0.0001, resampling).

Three different types of catheters were used for EVD insertion—HD-type, 306-type, and silver-impregnated AB-type catheters. In most cases—128 EVD insertions out of the total of 153 insertions—EVD drains impregnated with antimicrobial silver were inserted (84%). In the EVD-infected group, a 306-type EVD catheter was inserted in one patient (2%) and an HD-type EVD catheter in four patients (8%). In the non-infected group, 306-type EVD drains were inserted in eleven cases (11%) and HD-type catheters in nine cases (9%). There was no significant association observed between the AB (*p* = 0.1773, resampling) and HD (*p* = 0.5773, resampling) catheter type and EVD infection (Table 2). Nevertheless, for most of the patients (33 out of 37, 89.1%), the AB-type catheter was used.

### 2.4. Microbiology of EVD Infections and Other Systemic Infections

In the group with EVD infection, coagulase-negative *Staphylococcus* spp. (75%) were the most commonly isolated bacteria from the liquor. There were single patients who were infected with *Escherichia coli*, *Enterococcus feacalis*, *Moraxella* spp., and *Acinetobacter baumannii*.

In patients with an EVD, urinary tract infection was observed in 13 patients (35.13%) in the infected group and 40 patients (32.52%) in the group who did not have an infected EVD. Pneumonia was observed in 56.75% and 53.48% of the infected and non-infected groups, respectively, and wound infection in 5.4% and 4.65%, respectively.

In contrast, eight patients (21.62%) in the infected group had a bloodstream infection. In the non-infected group, bloodstream infection was observed in 11 patients (12.79%).

In the EVD-infected group, the rate of occurrence of multi-drug-resistant (MDR) bacteria (extended-spectrum beta-lactamase (ESBL)-producing Enterobacterales, vancomycin-resistant Enterococcus, methicillin-resistant *Staphylococcus aureus*, multi-drug-resistant *A. baumannii*) was 22% (eight patients), while in the non-infected group, MDR bacteria could be detected in 13% (eleven) of patients. In our study, methicillin-resistant *S. aureus* colonization was observed in five patients, ESBL-producing Enterobacterales colonization was detected in six patients, and vancomycin-resistant Enterococcus colonization was noticed in a number of EVD-infected patients.

### 2.5. Antibiotic Treatment

Patients with an EVD procedure received periprocedure antibiotics if the EVD placement took place in the OR concurrently with another operation that took place at the same time. In case the patients needed only EVD placement, it was performed bedside in the ICU, and patients did not receive antibiotics prophylaxis. In case an EVD infection was diagnosed, patients received a combination of vancomycin and meropenem therapy systemically due to the good central nervous system penetration of both antibiotics. One patient with EVD infection caused by multi-drug-resistant *A. baumannii* received not only systematic but also intrathecal colomycin therapy. In the case of an EVD infection, the patients were treated with systemic meropenem and vancomycin. Patients with EVD (both in the infected and non-infected groups) received other types of antibiotic treatment based on the antibiotic sensitivity of the bacteria that were responsible for their systemic respiratory tract or urinary tract infections. In case of pneumonia or urinary tract infection, the patients were treated with piperacillin/tazobactam, imipenem/cilastatin, ceftriaxone, or levofloxacin antibiotics. In case of aspiration pneumonia, clindamycin was used. In the case of multi-drug-resistant *A. baumannii* infection, the patient received systemic or intrathecal colomycin treatment.

### 2.6. Variable Importance

**Figure 4:**
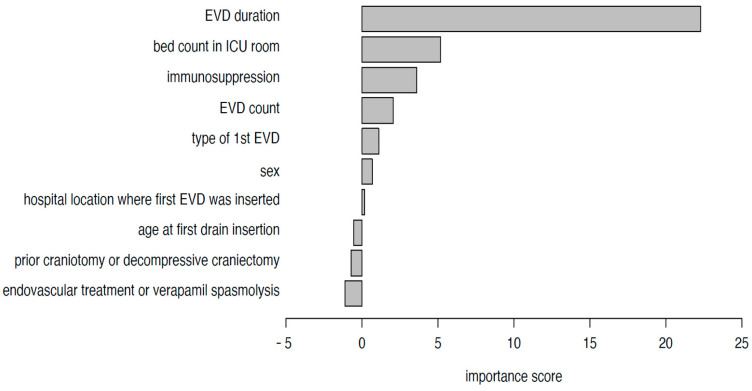
Variable importance for EVD infections.

Our limited sample size did not enable the quantification of the role of potential predictors using a multivariable model. Instead, we used the random forest method to assess variable importance: we found that the total duration of EVD was by far the most important predictor; other important predictors included bed count in the ICU room and immunosuppression status (Figure 4).

## 3. Discussion

Several studies describe numerous risk factors, including patient specifics and drain characteristics, that influence the development of EVD infections. Age and gender were identified as a risk factor for EVD infection, with indications that female patients are more likely to have an EVD infection than male patients [18,19,20]. In our study, we similarly observed a higher ratio for female patients, albeit to only a moderate extent. Moreover, in our study, prior craniotomy failed to show any association with the development of EVD infections; furthermore, neither SAH nor ICH was associated with EVD infection. This finding is to some degree in contrast to those of others, because earlier brain surgery has been demonstrated to be an independent risk factor for EVD infection [3,5,6,8]. Co-infection, as a risk factor with a higher incidence among patient factors, has been identified and found to be significantly associated with ventricular catheter infection [14,21,22,23,24]. Indeed, we observed a higher prevalence of urinary tract infections and bloodstream infections that were linked to the presence of an EVD infection. The likely reason for this finding is the prolonged ICU stay rather than a close association with EVD.

In our study, we managed to confirm the presence of catheter-related risk factors in the background of EVD infections. Previous retrospective studies have focused on EVD-related infections, where the EVD was placed inside the OR or outside of it, including in the ICU. In these retrospective studies, controversial results were observed [24,25,26,27]. One study described a higher infection rate following insertion in the OR, but this was attributed to the healthcare worker [24]. Another study reported a higher EVD infection rate following insertion in the OR, but this was due to the use of a cranial bolt kit brought from the OR next-door to the ICU [25]. In contrast, two other studies observed a higher infection rate following insertion in the ICU compared with insertion in the OR, but these data do not yield statistical significance [26,27]. In our case, insertion at the ICU similarly involved the use of a cranial bolt kit from the OR with the assistance of OR staff, and there was no difference observed in EVD infection rates with respect to the site of insertion, OR or ICU.

Earlier studies have demonstrated the impact of the dissemination of multi-drug-resistant bacteria on multiple-bed rooms in the ICU [28]. To the best of our knowledge, we investigated for the first time the extent to which EVD infection following EVD insertion in the ICU is influenced by the number of beds in the room. According to our results, the rate of EVD infections was much lower after insertion happened in a single-bed room compared to two- or five-bed rooms. However, there was no statistical difference in EVD infection based on whether the placement took place in a two- or five-bed ward. These observations could be related to environmental factors. There are multi-bed wards in low- and middle-income countries, so based on our results, the importance of environmental factors arises; therefore, additional infection control measures for hand hygiene, strict aseptic conditions, and environmental microbiological surveillance are justified in case of EVD infections.

Even though a limit for dichotomization (14.5 days) was determined on mathematical grounds, this number should not be considered a strict cut-off value but only a rule of thumb, since the change in infection frequency is gradual. Moreover, cut-off points should not be generally determined on purely mathematical grounds, as clinical substantiation is also necessary and frequently more valuable.

The EVD catheter type is also a risk factor associated with ventricular catheter infection. Antimicrobe-impregnated catheters (including antibiotic-impregnated and silver-impregnated catheters) have the potential to reduce infection in patients requiring an EVD. In our study, silver-impregnated catheters and plain catheters were in use; however, an antibiotic-coated catheter was never used. Silver-impregnated EVD catheters appear to be effective in reducing the risk of EVD infections, as found by many studies. Keong et al. reported that patients with silver-impregnated catheters have a lower risk of developing infection [29]. Three cohort studies also reported that silver impregnation reduces the infection rate [30,31,32].

EVD infection can result from contamination from the skin during EVD placement [1,21,33,34]. It may also be introduced secondarily to frequent EVD manipulations [2]. Prolonged EVD duration is also associated with an increased risk of EVD-related infection [2,5,13,34]. The duration of catheterization was identified as a risk factor by many authors, and the duration of catheterization ranged from 1 to 44 days in these studies [1,11,13,18,33,35]. Some authors suggest that a longer duration of catheterization may induce microbial infection of the catheter [1,21,33,34]. Most studies report a lower EVD infection rate in the first 5 days of EVD drainage and document that the infection rate increases significantly after 5 to 10 days of EVD placement [1,21,26]. Lozier et al. observed an increasing daily infection rate, with the peak around the 9th and 11th days [2]. Meanwhile, Paramore et al. detected promptly increasing daily infection rates [33]. Our study presented a similar result, as the EVD duration showed a correlation with EVD infection. However, in contrast to other studies, where the rate of infection increases significantly 5–10 days after insertion, in our case, the infection increased significantly on the 15th day after EVD insertion. Our results were ambiguous in terms of EVD infection rates. Despite regular CSF sampling every five days in all EVD patients, the daily EVD infection rates increased significantly only after day 15 and showed only a slow decrease. We hypothesize that the reason for this observation was due to the inappropriate CSF sampling practice. In our department, until now, CSF sampling was routinely carried out every fifth day in patients with EVD, and in the case of infection, every third day in order to closely monitor the patient’s condition. Despite the very strict aseptic conditions used during EVD insertion and sampling, the increased frequency of sampling may have contributed to the infections.

The use of multiple catheters was also identified as a risk factor for EVD infection [20,26,36]. Arabi et al. and Peter et al. report infection rates of 42% and 84.5% in patients who received multiple catheters vs. 3% and 18.3% in patients who did not, respectively [26,36]. Lo et al. also reported that infected patients received almost twice as many ventricular catheters as their uninfected counterparts [20,26,36]. Each additional catheter is reported to increase the risk of infection by 4-fold or 8% [20,36]. Arabi et al. found out that antibiotics were given more frequently with the first insertion than with repeated insertions—68% vs. 7%—and this practice is responsible for making multiple catheters a risk factor [26]. These observations were also confirmed by our results, as insertion of multiple catheters could be observed in the infected group.

As demonstrated in previous studies, the most common pathogens associated with EVD infections are coagulase-negative staphylococci originating from the skin flora [5,6,7,8,11,34,37]. Other pathogens include *E. coli*, *Enterococcus faecalis*, *Morganella* spp., and *A. baumannii* and infections in case of hospital-acquired infections, as is already described in other studies [38,39,40].

During our study, one shortcoming can be identified: not fully appropriate medical practices. Several studies have confirmed the role of antibiotic prophylaxis in infection prevention. For EVD insertion, antimicrobial prophylaxis regimens can be exclusively periprocedural (only prior to or during insertion) [1,11] or may continue for the entire duration of EVD placement [41,42,43,44,45]. Given that the current protocol for EVDs being inserted in the ICU does not prescribe antibiotic prophylaxis, we believe that a change in the protocol may lead to a reduction in the number of infections.

The introduction of infection control measures is important to improve the processes of ventricular drain care and patient outcomes. It is known that in hospitals that have improved their infection control approach for EVD, the reported infection rates were less than 1% [46,47]. Good practices in bundling sterile technique, tunneling of the catheter, use of periprocedural antibiotics only, use of an impregnated catheter, use of a closed system, lack of routine CSF sampling, use of sterile dressings, and no position change after insertion can reduce EVD infections [10,11,46,48,49,50]. Based on our results, we introduce a bundled care approach for EVD and follow-up as a part of a prospective study Table 3 below.

## 4. Study Limitation

Due to the nature of the present study, various limitations must be mentioned. Our study was a single-center, retrospective cohort study with the usual shortcomings intrinsic to such a design, including a small patient pool and limited diagnostic data. The fact that this study was conducted at a single center limits the generalizability of the findings.

This observational study did not include a priori power analysis to establish an optimal sample size; therefore, it is possible that meaningful associations were neglected due to the confidence intervals/*p*-values. The main reason for this was the lack of an established clinically meaningful effect size. Therefore, the sample size was the amount of available data during the study period, and the main goal of our study was to explore and describe potential risk factors for EVD infections.

## 5. Materials and Methods

### 5.1. Study Design

In our retrospective cohort study, we investigated 123 patients’ data regarding 156 EVD insertions. The data were collected at the Department of Neurosurgery and Neurointervention, Semmelweis University, Budapest, Hungary—formerly the National Institute of Mental Health and Neurosurgery—during the time period from 1 January 2022 to 31 March 2024. The patient population covers non-traumatic neurosurgery and neurointerventions requiring acute care, and as the National Institute, the department is a central care provider for different central nervous system disorders—for example, tumors, epilepsy—requiring surgical treatment.

Descriptive analysis was used to summarize patient demographics, neurological diseases, surgical treatment, underlying diseases, and infections and to analyze the EVD characteristics (antimicrobial or plain catheter); the EVD insertion place (OR, ICU, or other); single or multiple EVD catheterizations; EVD duration time; and outcomes.

### 5.2. Infection Diagnosis

The Centers for Disease Control and Prevention (CDC) guidelines for defining CSF infection were used [51]. At least 1 of the following criteria had to be met: (1) positive CSF culture; (2) fever > 38 °C with no other recognized cause; and either an elevated CSF protein level (>40 g/dL) and hypoglycorrhachia (<45 mg/dL) or organisms found on CSF gram staining. Healthcare-associated infections were defined using the CDC/NHSN surveillance definitions [52].

### 5.3. EVD Infection Control Protocol

The EVD infection control protocol included the implementation of standard protocols for EVD placement and EVD manipulation in the ICU. During EVD insertion, complete hair removal, degreasing, and strict aseptic techniques—a sterile pre-assembled EVD package, a cranial bolt kit brought from the OR—were used. The EVD was inserted in the presence of an operating nurse after surgical washing by the personnel. The skin was prepared using a skin disinfectant containing propanol. During EVD insertion, a silver-impregnated EVD catheter (AB) (Sophysa, Rouen, France) was mainly used, or plain catheters (306 and HD) were used under special indications (such as massive hemorrhage). EVD dressing involves 3–5 cm of tunneling, and antimicrobial bandage (Wolf Ltd, Győr, Hungary) is a large clear dressing (Tegaderm, 3M, Budapest, Hungary) placed on the surgical thread to secure the catheter. Routinely, CSF samples were taken every fifth day from each patient and every third day in case of meningitis.

There was no special protocol for shortening the duration of the EVD, but as soon as the patient’s clinical condition allowed for it, the EVD was removed.

In January 2024, work in the ICU was reorganized, as regular environmental surveillance, enhanced hand hygiene monitoring, and regular hand hygiene training were introduced, and the cleaning protocol was rearranged. The patient HCW ratio changed from the previous ratio of 1:3 to 1:2. The same equipment was available for each bed.

### 5.4. Statistical Analysis

Primary data collection and organization were carried out using MS Excel. Collected data included infection status, sex, age at first insertion, ICU stay in days, bed count in ICU room, ward stay in days, Glasgow coma score (GCS), intubation status, diagnosis, hypertension status, diabetes status, immunosuppression status, prior craniotomy, prior decompressive craniectomy, endovascular treatment through the femoral artery, verapamil spasmolysis through the femoral artery, ventriculoperitoneal shunt, and drain characteristics (date of insertion and removal, place and duration of insertion, drain type, and whether it was an emergency insertion).

To assess the importance of potential risk factors for EVD infections, we used a random forest method. We included 10 predictor variables that we thought may potentially be risk factors: sex, age at first drain insertion, hospital location where first EVD was inserted, bed count in the ICU room, type of first EVD, total number of EVDs inserted, duration of EVD, immunosuppression, prior craniotomy or decompressive craniectomy, endovascular treatment, or verapamil spasmolysis through the femoral artery. In the mathematical model, we used 500 trees to fit the random forest model, and the variable importance was assessed using the mean change in prediction error across all trees after permuting each predictor variable. Results are shown in bar charts. We used version 4.7.1.2 of the random Forest R-package. To determine a cut-off point for the dichotomization of the EVD duration with regard to the EVD infection outcome, we analyzed the receiver operating characteristic (ROC) curve and maximized for the sum of sensitivity and specificity. We used the pROC R-package (version: 1.18.5).

Descriptive statistics for each variable were provided for the infected and non-infected patient groups, respectively; additionally, differences between these values were assessed by their confidence intervals (CIs) and tests for significance. Alpha was set at 0.05. For categorical variables, counts and proportions (total: patient count by infection status) were provided; CIs for proportion differences were determined using “method 10”, as recommended by Newcombe [53]; *p*-values were calculated using a resampling method with 10,000 bootstrap samples. For continuous variables, means and standard deviations (SDs) were provided; CIs and p-values were calculated using Welch’s *t*-test. All statistical analyses were carried out in R version 4.4.1 (14 June 2024) [54].

## 6. Conclusions

Reviewing EVD infections, especially at regular intervals, and clarifying the underlying risk factors are very important. This highlights the deficiencies in the local protocol and the need to strengthen infection control, as well as to identify local hygiene problems. Acknowledging these problems, introducing care packages, and modifying antibiotic stewardship by introducing periprocedure antibiotic prophylaxis will hopefully contribute to a reduction in infections.

As a result of the present study, an EVD bundle care package will be introduced in our institute, which prioritizes the recommendations regarding the reduction in EVD manipulations, appropriate hygienic conditions, and periprocedure antibiotic treatment.

## Figures and Tables

**Table 1:** Patient Demographics and Clinical Characteristics by Infection Status (EVD infection vs. No EVD infection).

Characteristics	EVD Infection		No EVD Infection		Difference (EVD Infection–No EVD Infection)			
	n = 37		n = 86					
	Count and % Mean and SD		Count and % Mean and SD		Point Estimate	95% CI		*p*-Value
**Gender (male)**	16	0.43	40	0.47	−0.03	−0.21	0.16	0.7654
**Age mean (SD)**	55.08	12.05	56.09	12.84	−1.01	−5.83	3.81	0.6767
**Intubated status**	20	0.54	52	0.60	−0.06	−0.25	0.12	0.5955
**Glasgow coma scale (GCS) at insertion**								
eye: mean (SD)	1.86	1.16	1.76	1.11	0.11	−0.34	0.56	0.6291
verbal (not intubated): mean (SD)	3.41	1.33	3.41	1.33	0.00	−0.80	0.80	1
motor: mean (SD)	3.41	1.91	2.84	2.06	0.57	−0.20	1.34	0.1428
total (not intubated): mean (SD)	11.47	2.37	11.26	3.37	0.21	−1.44	1.85	0.802
**Primary diagnosis**								
subarachnoid hemorrhage	32	0.86	66	0.77	0.10	−0.07	0.22	0.5229
intracerebral hemorrhage	7	0.19	18	0.21	−0.02	−0.16	0.15	0.8113
other intracranial hemorrhage	1	0.03	2	0.02	0.00	−0.06	0.12	0.843
occlusion or stenosis of (pre)cerebral arteries	1	0.03	11	0.13	−0.10	−0.19	0.02	0.5292
tumor	3	0.08	9	0.10	−0.02	−0.12	0.12	0.723
traumatic brain injury	0	0.00	0	0.00	0.00	−0.04	0.09	1
**Immunosuppressed status**	5	0.14	5	0.06	0.08	−0.03	0.23	0.4737
**Diabetes mellitus**	6	0.16	9	0.10	0.06	−0.06	0.21	0.5246
**Hypertension**								
present. treated	21	0.57	39	0.46	0.11	−0.08	0.29	0.5299
present. not treated	4	0.11	15	0.18	−0.07	−0.18	0.09	0.5233
**Prior craniotomy**	8	0.22	27	0.31	−0.10	−0.24	0.08	0.5094
**Prior decompressive craniectomy**	2	0.05	4	0.05	0.01	−0.07	0.13	0.845

**Table 2:** Characteristics of EVD catheterization and infection rates.

Variable	EVD Infection		No EVD Infection		Difference (EVD Infection–No EVD Infection)			
	n = 37		n = 86					
	Count and % Mean and SD		Count and % Mean and SD		Point Estimate	95% CI		*p*-Value
**Hospital location where first EVD was inserted**								
ICU	31	83.78%	67	77.91%	5.88%	−10.81%	18.93%	0.5667
operating room	5	13.51%	18	20.93%	−7.42%	−19.78%	8.77%	0.5384
brought from elsewhere	1	2.70%	1	1.16%	1.54%	−4.05%	12.70%	0.5003
hospital location where all EVDs were inserted								
ICU only	30	81.08%	67	77.91%	3.17%	−13.84%	16.82%	0.7127
ICU (+else)	33	89.19%	70	81.40%	7.79%	−7.69%	19.32%	0.5338
operating room only	4	10.81%	16	18.60%	−7.79%	−19.32%	7.69%	0.5318
operating room (+else)	6	16.22%	18	20.93%	−4.71%	−17.69%	11.88%	0.6224
from other hospital only	0	0%	0	0%	0%	−4.28%	9.41%	1
from other hospital (+else)	1	2.70%	1	1.16%	1.54%	−4.05%	12.70%	0.5003
**EVD count**								
1	25	67.57%	75	87.21%	−19.64%	−36.66%	−4.18%	0.5027
2	9	24.32%	8	9.30%	15.02%	1.45%	31.45%	0.4852
3	2	5.41%	3	3.49%	1.92%	−5.47%	14.43%	0.6437
4	1	2.70%	0	0%	2.70%	−2.12%	13.82%	0.293
2–4	12	32.43%	11	12.79%	19.64%	4.18%	36.66%	0.5062
emergency EVD at any time	6	16.22%	5	5.81%	10.40%	−0.71%	25.68%	0.501
**EVD duration (days)**								
first EVD (only one EVD)	19.48	6.55%	11.09	8.315	8.38%	5.12%	11.64%	0
first EVD (one or more EVD)	20.35	12.98%	10.61	8.67	9.73%	5.05%	14.41%	0.0001
all EVDs combined	27.56	19.37%	12.63	10.13	14.92%	8.14%	21.71%	0.0001
**Duration of hospital stay (days)**								
ICU stay	27.86	17.06%	13.43	10.75%	14.43%	8.33%	20.53%	0
ward stay	8.16	9.46%	5.25	8.21%	2.9%	−0.67%	6.48%	0.1097
total hospital stay (ICU + ward)	36.02	17.56%	18.68	14.74%	17.34%	10.74%	23.93%	0
**Bed count in ICU room**								
1	3	8.11%	20	23.26%	−15.15%	−26.43%	0.11%	0.0847
2	15	40.54%	25	29.07%	11.47%	−6.08%	29.58%	0.3004
5	19	51.35%	41	47.67%	3.68%	−14.97%	22.00%	0.8591
2–5	34	91.89%	66	76.74%	15.15%	−0.11%	26.43%	0.0847
**Drain type**								
AB only	33	89.19%	66	76.74%	12.45%	−3.43%	24.35%	0.1773
AB (+other)	34	91.89%	69	80.23%	11.66%	−3.30%	22.66%	0.18
306 only	0	0.00%	8	9.30%	−9.30%	−17.30%	1.13%	0.1285
306 (+other)	1	2.70%	11	12.79%	−10.09%	−19.05%	2.32%	0.1621
HD only	2	5.41%	9	10.47%	−5.06%	−14.18%	8.17%	0.5773
HD (+other)	4	10.81%	9	10.47%	0.35%	−10.17%	15.07%	1

**Table 3:** Recommended infection control measures for EVD insertion.

Infection Control Measures	Current Practice	Action Necessary
Location of EVD insertion	OR or ICU bedside	No action
Aseptic condition at insertion	Strict aseptic condition at insertion	No action
Standardized dressings and weaning t	Excilon™ AMD Antimicrobial Dressings: polyhexamethylene biguanide hydrochloride-treated dressing 3M™ Tegaderm™ Transparent Film Dressing Frame Style	No action
Periprocedure antibiotic prophylaxis	Used in OP Not used in the ICU	Antibiotic prophylaxis must be introduced in ICU
CSF sampling frequnecy	no infection: every five days infection: every three days	Sampling only in clinically adequate cases
EVD properties	-primarily silver-impegrated AB-EVDs in use -special indications: non-AB-EVD in use	No action
EVD removal	Removed as early as possible	No action
Changing catheter sites	No routine catheter site change	No action
EVD insertion site	ICU bedside in multiple-bed rooms	ICU bedside in single-bed room
Environmental sampling in ICU rooms	Not carried out	Regular environmental sampling Regular air sampling

## Data Availability

The datasets used and/or analyzed during the current study are available from the corresponding author.
